# Bile aspiration during emergent revision of a single-anastomosis gastric (SASI) bypass to a Roux-en-Y configuration with transit bipartition: a case report

**DOI:** 10.1093/jscr/rjag786

**Published:** 2026-09-02

**Authors:** María Emilia Salgado, Carolina Guevara, Gabriel Carvajal, Gabriela Zapata Jaramillo, Napoleón Salgado Macías

**Affiliations:** Clínica Bariátrica Dr. Napoleón Salgado, Av. Mariana de Jesús y Nuño de Valderrama, 170509, Quito, Ecuador; Clínica Bariátrica Dr. Napoleón Salgado, Av. Mariana de Jesús y Nuño de Valderrama, 170509, Quito, Ecuador; Clínica Bariátrica Dr. Napoleón Salgado, Av. Mariana de Jesús y Nuño de Valderrama, 170509, Quito, Ecuador; Clínica Bariátrica Dr. Napoleón Salgado, Av. Mariana de Jesús y Nuño de Valderrama, 170509, Quito, Ecuador; Clínica Bariátrica Dr. Napoleón Salgado, Av. Mariana de Jesús y Nuño de Valderrama, 170509, Quito, Ecuador

**Keywords:** SASI bypass, bile reflux, transit bipartition, bariatric revisional surgery, pulmonary aspiration, gastroesophageal reflux disease

## Abstract

Single anastomosis sleeve ileal (SASI) bypass is an emerging bariatric procedure; some patients face complications such as severe bile reflux, requiring conversion. In this context, the surgical goal is to substantially reduce exposure of the stomach and gastroesophageal junction to bile acids to prevent early complications. We present a 46-year-old man with class III obesity (body mass index 54.6 kg/m^2^) who 10 days after an uneventful SASI bypass, developed intense, persistent, refractory reflux. A diagnostic endoscopy revealed an extensive accumulation of biliary reflux in the gastric lumen, prompting reintervention. During anesthetic induction, the patient aspirated bile, causing acute respiratory distress. After intraoperative stabilization, the team proceeded without further incident, converting the single-loop configuration to a Roux-en-Y transit bipartition, resolving gastroesophageal reflux disease (GERD). This case underscores the need to incorporate prevention maneuvers during anesthetic induction in patients with severe GERD to avoid aspiration, and the importance of anticipating high anesthetic risk in these individuals.

## Introduction

Single anastomosis sleeve ileal (SASI) bypass is a modification of sleeve gastrectomy with transit bipartition (the Santoro procedure) that creates a single loop anastomosis rather than a Roux-en-Y double anastomosis [1]. This configuration can produce intense distal gut stimulation, reduce proximal bowel activity, enhance satiety, and decrease meal size [1]. However, the one-loop arrangement can predispose to free reflux of bile into the stomach, promoting gastroesophageal reflux disease (GERD) that may require re-intervention to resolve [2, 3].

Reports on this complication are heterogeneous: symptomatic biliary reflux occurs in 0.5%–3.4% of cases [4], and, when diagnosed by endoscopy, biliary reflux has been reported in 62.9% of post-SASI patients versus 11.4% preoperatively [5]. For the surgical management of refractory reflux, conversion to a Roux-en-Y configuration is reported as the main option; two cases resulting in remission of reflux have been reported [6, 7].

We present an early biliary reflux complication 10 days after a SASI bypass; its severity and intensity led to aspiration of biliary content during conversion to a Roux-en-Y transit bipartition (Santoro procedure).

## Case report

**Figure 1:**
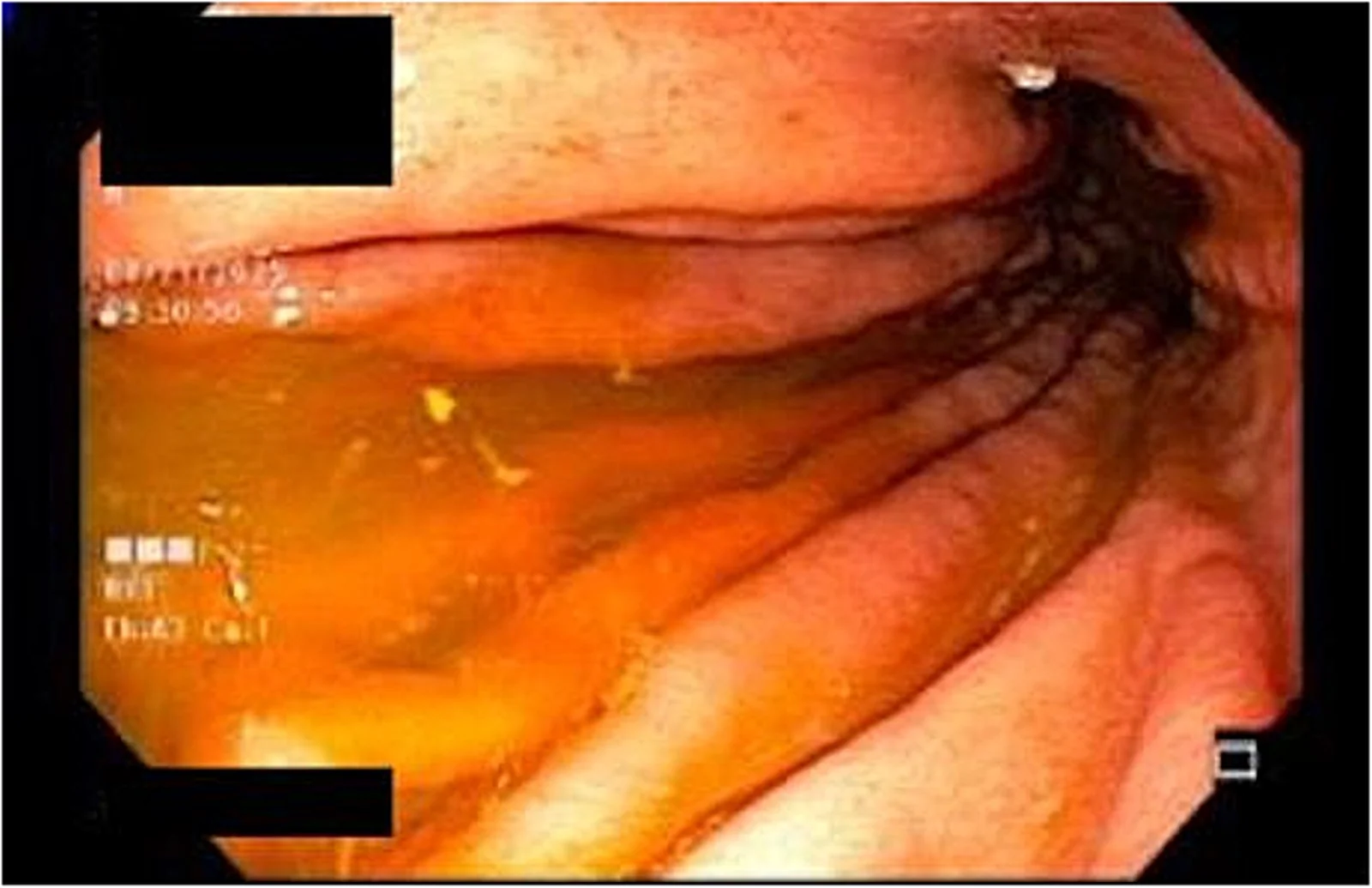
Baseline upper gastrointestinal endoscopy before SASI bypass: gastric lumen without significant reflux.

A 46-year-old man with class III obesity [body mass index (BMI) 54.6 kg/m^2^], no prior history of GERD, hypertension (150/88 mmHg), insulin resistance (HOMA-IR 14.31), hyperuricemia (7.9 mg/dl), and hepatic steatosis was admitted for an elective SASI bypass. A baseline upper endoscopy before surgery showed a gastric lumen without significant reflux (Fig. 1). The procedure was performed without complications; a blue dye test confirmed limb permeability and no leakages. The patient was discharged on postoperative day 2 with general care and nutritional guidance.

Seven days after surgery, the patient reported persistent nocturnal gastroesophageal reflux requiring head-of-bed elevation for relief, with episodic vomiting. Daily omeprazole and meal-timing changes were prescribed, with close follow-up. By day 10, reflux became severe and refractory to medication; the patient could not tolerate liquids, continued vomiting, and reported a sensation of suffocation and choking. On examination, the tongue appeared bile-stained (yellow).

**Figure 2:**
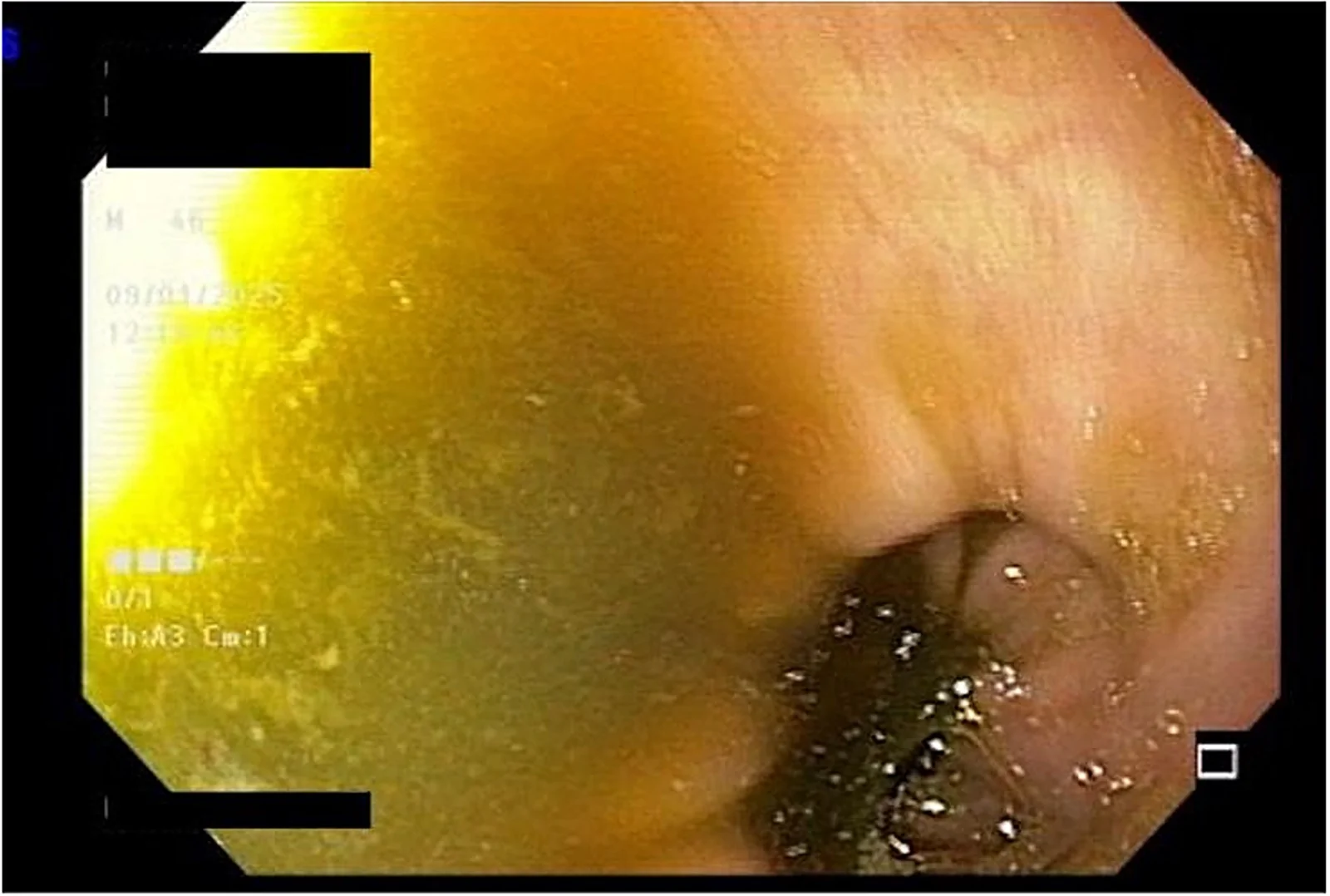
Control upper gastrointestinal endoscopy 10 days after SASI bypass: gastric lumen with significant reflux.

**Figure 3:**
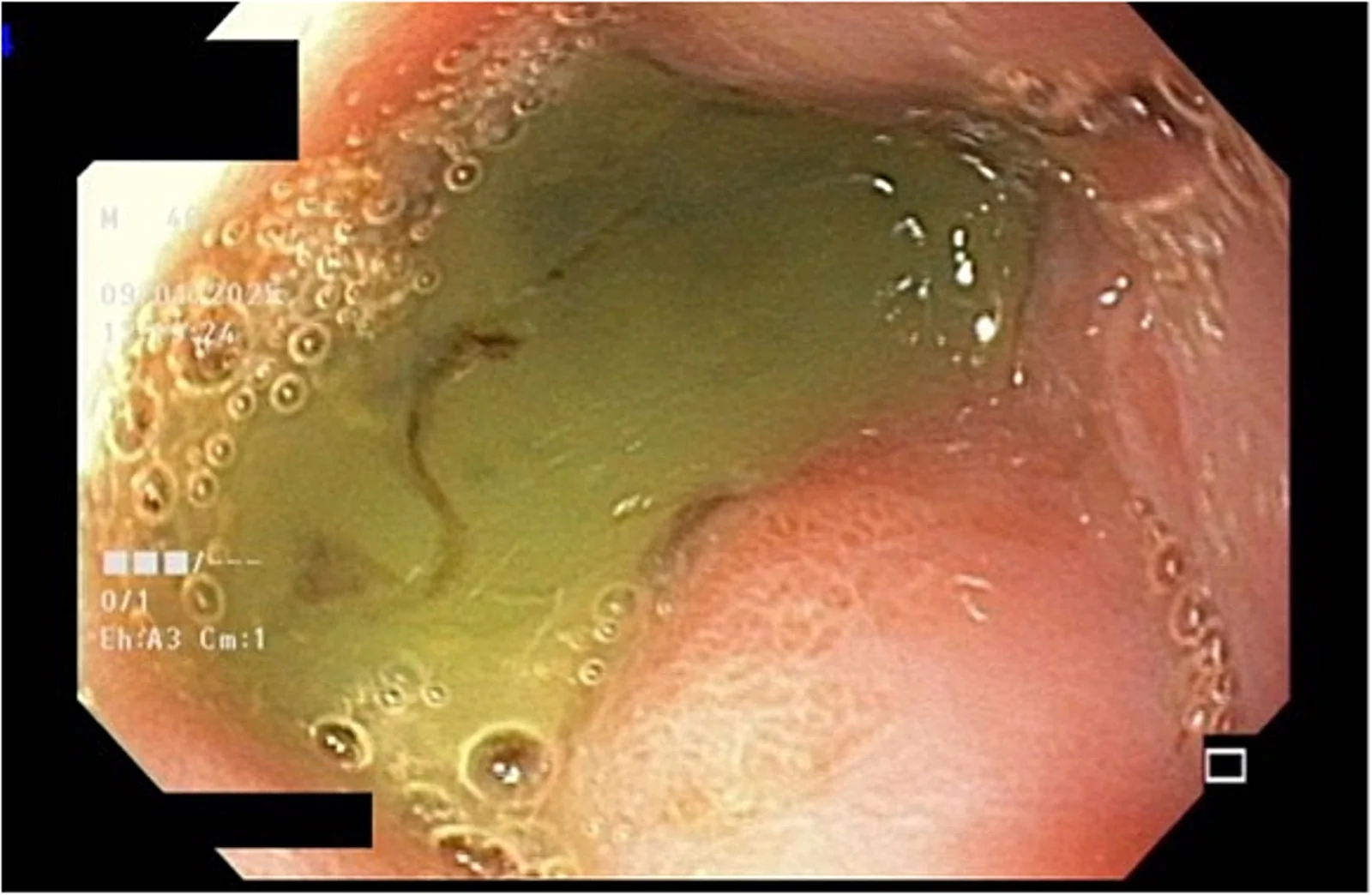
Control upper gastrointestinal endoscopy 10 days after SASI bypass. Pooling of abundant bilious reflux content within the gastric remnant.

Diagnostic preoperative upper endoscopy confirmed severe GERD, with intestinal fluid reflux at the esophagogastric junction and extensive intragastric accumulation (Figs 2 and 3). Re-intervention was decided, and a Roux-en-Y transit bipartition (Santoro procedure) was planned. Anesthetic evaluation determined an ASA III status, and induction was performed with remifentanyl and propofol, followed by rapid sequence induction (RSI) protocol. However, owing to excessive biliary gastric content, the patient aspirated before the endotracheal tube could be secured.

After intraoperative stabilization, the conversion was completed without further complications: the afferent (biliopancreatic) limb was divided close to the gastroileal anastomosis with a linear stapler and re-anastomosed distally in a Roux-en-Y configuration with a 50-cm alimentary limb, diverting biliopancreatic flow away from the sleeve while preserving the duodenal route (transit bipartition); the intestinal defect was closed with Vicryl sutures.

The patient was admitted to the intensive care unit (ICU) with severe acute respiratory distress syndrome (ARDS) (Berlin criteria; PaO₂/FiO₂ 86), type I acute respiratory failure, and aspiration pneumonia. With ventilatory support and antibiotic therapy, respiratory improvement began after 3 days, and ventilatory weaning was initiated. By day 4 in the ICU, the patient no longer required mechanical ventilation and continued to improve. By day 9, pneumonia had resolved, and supplemental oxygen was no longer needed. By day 11, he was discharged without complications and remained on close follow-up.

Eight months after the conversion, the patient remains free of GERD symptoms, and metabolic targets have been met: insulin levels have normalized, with 25% total weight loss (TWL) and 46.1% excess weight loss (EWL), and a BMI of 40.9 kg/m^2^.

## Discussion

The SASI bypass, described by Tarek Mahdy in 2016 [1], is indicated for patients with high BMI and is designed to induce an early incretin stimulus in the distal intestine, thereby achieving greater satiety regulation and weight loss than a conventional gastric bypass. This configuration also allows easier access to the first portion of the duodenum [8]. These criteria, along with the relatively lower surgical complexity of the SASI bypass, which requires only one loop anastomosis, led the surgeon to consider it the most suitable technique for our patient.

Nevertheless, this configuration can promote bile and pancreatic enzymes to reflux more easily toward the created sleeve [9]. The intensity and severity of this reflux can reach the esophagus, producing a refractory, symptomatic complication that indicates re-intervention. The most effective in ensuring adequate bile drainage is Roux-en-Y bypass [5, 10].

In this case, converting to a Roux-en-Y transit bipartition diverted the biliopancreatic limb away from the sleeve, key to eliminating bile reflux. Conversions of this type, by reducing biliary exposure of the sleeve, have been associated with short-term GERD remission, effective metabolic regulation, and weight loss [11]. Reported cases of resolution of severe GERD after revisional conversion to a Roux-en-Y configuration support this approach [6, 7]. To our knowledge, this is one of the first reported cases of an urgent conversion from a SASI bypass to a Roux-en-Y transit bipartition (Santoro procedure) in Ecuador.

The most striking aspect of this case was the bile aspiration during anesthetic induction. Extensively accumulated intragastric bile, the source of reflux for 10 days, constituted the aspiration risk that led to severe ARDS (PaO₂/FiO₂ 86). This underscores the importance of categorizing bariatric patients with severe GERD as high risk for intubation. The combination of a full gastric remnant and an incompetent gastroesophageal junction warrants additional precautions during anesthetic induction [12, 13].

This case illustrates the value of early reintervention in patients with severe reflux, before nutritional and clinical deterioration increases anesthetic risk. Patients with high BMI and severe reflux should be assessed anesthetically as a full-stomach, high-aspiration-risk case (analogous to high small-bowel obstruction). Pre-surgical nasogastric decompression is an option to evacuate the bile and prevent aspiration. In this case, we did not consider this option. Blindly introducing a gastric tube could pose a risk of staple line injury or gastric sleeve perforation. Also, awake fiberoptic intubation is an alternative to rapid-sequence intubation that may reduce the risk of aspiration [13–15].
